# A narrative review of the application of the survey experiment in sports psychology: research design and method

**DOI:** 10.3389/fpsyg.2025.1631244

**Published:** 2026-01-07

**Authors:** Chenping Zhang, Zhiguang Ji, Liyan Wang, Hongbiao Wang

**Affiliations:** 1 Department of Physical Education, Shanghai University of Medicine and Health Sciences, Shanghai, China; 2 College of Rehabilitation Science, Shanghai University of Medicine and Health Sciences, Shanghai, China

**Keywords:** survey experiment, sports psychology, survey, experiment, research validity

## Abstract

**Introduction:**

As a research paradigm integrating internal and external validity, the survey experiment method has been widely used in the humanities and social sciences. However, there is currently no research that provides a comprehensive summary of the application of survey experiment in sports psychology.

**Methods:**

In present study, we conducted a literature search of PubMed, Cochrane Library, Web of Science, ELSEVIER and EBSCO from January 1st, 2001 to April 30th, 2025, using the following search terms of titles and abstracts: “Survey Experiment.”

**Results:**

In total, 59 studies were included in the final review, after thoroughly reviewing all the studies, the current review addressed the following aspects using narrative method: the connotations and characteristics of survey experiment; the design of survey experiment; a case study on the design of survey experiment in sport psychology research; and the advantages of the Internet and big data in advancing survey experiment.

## 1 Introduction

In scientific research, validity pertains to the reliability and accuracy of a study’s results. Researchers frequently employ validity as a criterion to evaluate research quality (Ahmed and Ishtiaq, 2021). Validity consistently plays a pivotal role in every stage of research, from study planning and experimental design to critical analysis of research reports (Sürücü and Maslakci, 2020).

In academic research, validity can be divided into two main categories: internal and external (Slack and Draugalis, 2001). Internal validity concerns the internal factors within a study that influence the results or their interpretation. It is achieved when there is a single interpretation of the results, which are compromised by any factors that give rise to alternative interpretations (Halperin et al., 2015). In contrast, external validity refers to the extent to which study results remain valid outside the study’s constraints (Druckman et al., 2011). Can these results be applied to other populations, contexts, or measurements? Some research situations raise doubts about the generalizability of the results, as research results are commonly generalized from tightly controlled laboratory settings to the uncontrolled real world (Onwuegbuzie, 2000). The challenge of generalizing findings beyond a specific study pertains to external validity. A study possesses external validity if its results are equally applicable to diverse times, situations, and environments outside the specific experimental scope, reflecting the true relationships between the variables. Conversely, a study lacks external validity if its results are only valid within the constraints defined by the study (Baldwin, 2018).

The results derived from a tightly managed and well-observed experimental setup may not be able to replicate the complexities of the real world (Schmuckler, 2001). Data collected in an environment that is meticulously structured and overseen may not be transferable to the unpredictable conditions of the everyday world. Outcomes acquired in a highly organized, strictly monitored laboratory environment may not be applicable to unpredictable conditions (Kihlstrom, 2021). The need to explore cause and affect relationships has become an important issue in the social sciences. A key example of this was the 1898 study by Triplett (1898), an American social psychologist, on the ‘social facilitation effect’, where he showed that cyclists performed faster when they were accompanied by others. This was a significant development in the field of social science research, leading to the emergence of experimental sociology, experimental political science, and experimental economics. The experimental research paradigm is currently the dominant method used in social science research (Morton and Williams, 2010).

In the social sciences, experimental studies are usually classified into laboratory, field, and natural experiments (Diamond, 1983). Laboratory experiments use random allocation of samples to try to balance and control confounding variables, but the cost and sample size limit the ability to completely balance the effects of these variables (Falk and Heckman, 2009). Field experiments simulate naturally occurring scenarios, which improves the study’s external validity but at the cost of internal validity (Baldassarri and Abascal, 2017). Natural experiments have the least control over environmental and material variables, making it difficult to manipulate the target variable of stimulation; thus, they have limited application in the social sciences (Titiunik, 2021). Cross-sectional surveys are a common tool in social science research, but they address a host of potential issues. Selective bias, spurious correlation, correlation measurement error, censored data, and lack of true reciprocal causality are all dangers associated with inferring causality from cross-sectional survey data (Spector, 2019). To reduce bias, researchers may employ techniques such as matched designs, propensity scores, and two-stage least squares (Kirk, 2009). Panel surveys, which collect data from the same individuals over multiple time periods, can also be used to examine longitudinal changes. However, they are expensive and difficult to implement and are subject to the same limitations as cross-sectional surveys, as well as the exercise effect, which can distort survey results due to repeated surveys of the same individuals (Kalton and Citro, 1995).

Despite the significance of the experimental research paradigm in the social sciences, survey research continues to be the predominant method, especially in sociology, political science, and demography, due to its robust external validity and its ability to accurately estimate population parameters using large samples (Kent, 2020). Survey research seeks to establish a correlation between two variables without delving into causation. It typically observes and records two naturally occurring variables without any manipulation, control, or intervention, thus ensuring high external validity (Adam, 2020). However, the lack of control or monitoring of variables in the study facilitates the introduction of additional variables (potential confounding variables), consequently reducing internal validity.

In summary, it is challenging for commonly used experimental research and survey studies to balance internal and external validity. The literature review revealed that survey experiment integrates the advantages of survey research, including large random samples for external validity, with the benefits of experimental research, such as control and manipulation for internal validity (Martini and Olmastroni, 2021). However, there is currently no research that provides a comprehensive summary of the application of survey experiment in sports psychology. Therefore, this review aims to address this gap by systematically reviewing the literature on the use of survey experiment in the field of sports psychology.

## 2 Methods

### 2.1 Search strategy

We conducted a literature search of PubMed, Cochrane Library, Web of Science, ELSEVIER and EBSCO from January 1st, 2001 to April 30th, 2025, using the following search terms of titles and abstracts: “Survey Experiment.” Articles in review articles were manually searched for potential inclusion.

### 2.2 Study selection

**Figure 1:**
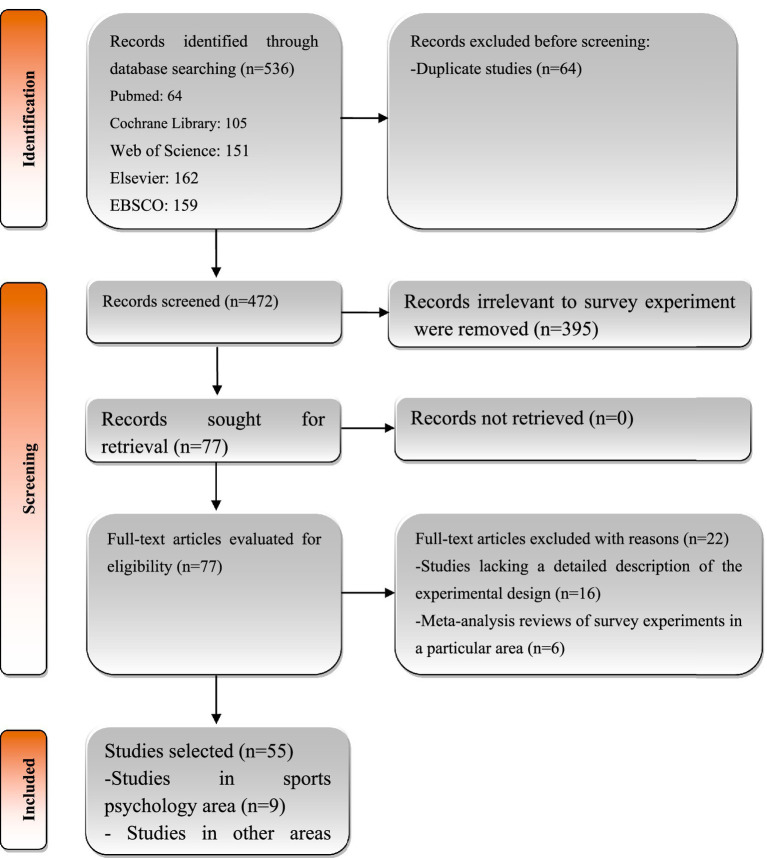
Flow chart of literature review.

The studies selected included those with the following criteria: a study published in English in a peer-reviewed journal; the studies included for section 3.1, and 3.2 had no limitation in research area; the studies selected for section 3.3 should focus on sports psychology area. The flow diagram of the selection process please sees Figure 1.

### 2.3 Quality appraisal

Quality appraisals were first performed by reviewer one (Ji ZG) and was subsequently checked by the second reviewer (Zhang CP). Where queries or concerns arose, consensus was achieved between the two reviewers. The screening process for the articles began and initial title screening was conducted by two independent reviewers (Wang LY and Wang HB), then followed by full text review of potentially eligible studies by the other two reviewers (Zhang CP and Ji ZG).

### 2.4 Data analysis

This review is a narrative synthesis review based on a literature review. First, we outlined the framework of this review based on a thorough literature review of included studies; Then, we carefully reviewed the included studies to extract information related to the previous step. For the content of section 3.2, four authors identified representative cases from the eight included articles. Finally, we summarize and synthesize the findings into this review.

## 3 Results

Fifty-five studies were included in the final analysis. The main countries the studies conducted including: United State, Germany, Italy, and the United Kingdom, Norway, Argentina, China, India, Russia, Spain, Japan, Italy, Brazil, Korea while one study collected data from around 35 developing countries. Based on a thorough literature review, the current review would address the following aspects: the connotations and characteristics of survey experiment; the design of survey experiment; a case study on the design of survey experiment in sport psychology research; and the advantages of the Internet and big data in advancing survey experiment (Table 1).

**Table 1:** The characteristic of included studies.

Section	Theme	Summary of findings	Main included studies
3.1	The connotation and characteristics of survey experiment	The main characteristics of survey experiment research including: large sample size; samples can be randomly selected to be broadly representative, and large random samples minimize differences between groups of participants so that even weak effects between treatments levels can be detected; the ability to discern sequential cause-and-effect relationships through experimental design; the unique intervention methodology.	Avidit et al. (2018), Barabas and Jerit (2010), Bremer and Bürgisser (2023), Carron et al. (2002), Knudsen and Johannesson (2019), Ghassim et al. (2022), Gong and Wang (2021), Horiuchi et al. (2007), Hyde (2015), Kirk (2009), Lee et al. (2025), Martini and Olmastroni (2021), Meng et al. (2014), Mummolo and Peterson (2018), Mutz (2011), Samuels and Zucco (2014), Shadish et al. (2001), Schlueter and Schmidt (2010), Sheffer et al. (2018), Sniderman (2018), Druckman et al. (2011), Sungmin et al. (2025), Thyer (2010), Wallander (2009), Watts, (2017), and Xie and Dong (2021)
3.2	Design of the survey experiment	This section emphasized the design of the survey experimental, which involves three parts: Manipulation of variables in survey experimental research. In this part, we elaborated on the research designs that are commonly employed for variable manipulation in survey experiments; Randomized sample allocation; Random sample selection.	Araújo et al. (2019), Auspurg and Hinz (2014), Acharya et al. (2013), Bansak et al. (2021), Braga et al. (2017), Furnham and Boo (2011), Glynn (2013), Gobo (2004), Keren and Lewis (1993), Lei and Lu (2017), Molden (2014), Mullinix et al. (2015), Ngoye et al. (2020), Raab et al. (2019), Ried et al. (2022), Schiff et al. (2022), Treischl and Wolbring (2022), and Webster and Sell (2014)
3.3	The application of survey experiment in sports psychology	In this section, we presented three typical cases of survey experiment applied in the field of sports psychology. Through these three cases, we demonstrated how survey experiment can be applied in the field of sports psychology.	Buckworth and Dishman (2007), Chang and Krosnick (2010), Dawson and Dobson (2010), Jones et al. (2002), Lane et al. (2006), Mazzeo et al. (2018), Molden (2014), Morente-Sánchez and Zabala (2013), and Williams and Jackson (2019).
3.4	The advantages of the internet and big data in fueling survey experimental research methods	The impact of the internet and big data on the research paradigm in sport psychology is reflected in four aspects: Expands the sample space; The experimental context aligns closely with real-life scenarios; Enhancing the study’s external validity and facilitating the exploration of interaction effects; Help mitigate complexities resulting from an excessive number of factors.	Walker et al. (2017), and Walker et al. (2019).

### 3.1 The connotation and characteristics of survey experiment

Survey experiment has emerged as a central methodology in the social sciences (Mullinix et al., 2015). They combine the strengths of survey research, such as large sample random sampling for external validity, with those of experimental research, including control and manipulation for internal validity (Martini and Olmastroni, 2021). This approach reveals the causal relationships between social phenomena and elucidates the essence and developmental patterns of the experimental object (Sniderman, 2018). By integrating the traditional questionnaire with experimental logic and conducting experiments on experimental subgroups with a certain degree of reliability and validity, this method generates results that can be generalized to a wider population. Initially, survey experiment surfaced in Western political science, particularly in public opinion polling, administration, public governance, and other focal areas of political science (Gong and Wang, 2021; Wallander, 2009; Watts, 2017); subsequently, several noteworthy classic survey experiment research cases were conducted (Avidit et al., 2018; Barabas and Jerit, 2010; Knudsen and Johannesson, 2019; Horiuchi et al., 2007; Hyde, 2015; Meng et al., 2014; Mummolo and Peterson, 2018; Mutz, 2011; Samuels and Zucco, 2014; Schlueter and Schmidt, 2010; Sungmin et al., 2025).

Similar to a traditional questionnaire, a survey experiment research involves a paper or electronic survey that prompts respondents to answer a series of questions regarding the issue under study, presented in textual or other formats. However, unlike traditional questionnaires, survey experiment involves two or more versions of the questionnaire, which are randomly assigned to the survey respondents (subjects) using methods such as alternating single and double numbering of the questionnaire codes (Xie and Dong, 2021). The primary distinguishing features of survey experiment, in contrast to traditional questionnaires, are randomization and external intervention (Shadish et al., 2001).

The aim of the experimental design is to randomly select a sizable subject sample, allocate it into diverse experimental units, control for confounding variables, manipulate independent variables, and measure dependent variables to investigate the impact of the independent variables on the dependent variables, ultimately establishing a causal relationship between the response and target variables (Kirk, 2009).

In research designs where the target variable is not manipulated but merely a change in its value is observed, the study can explore only the correlation between the target variables, not causation. Manipulating the target variable without controlling for confounding variables constitutes a pre-experimental study, such as a single-group post-test design with non-randomly selected subjects, no control group, and a single experimental treatment with one post-test (Thyer, 2010). Another example is the one-group pre-post test design, which contains an additional pre-test in which the post-test is subtracted from the pre-test to measure the effect of the experimental treatment, implying limited internal validity (Thyer, 2010). When the target variable is manipulated and confounding variables are controlled but random assignment is not feasible, this constitutes a quasi-experimental study, such as a nonrandomized pre-post test control group design with experimental and control groups not randomly assigned. The differences between groups can affect the causal relationships between the target variables. If random assignment is possible, the study is considered a true experiment. Despite convenient sample selection, many true experiments in the social sciences, including political science, sociology, and economics, has integrated survey research, known as survey experiment research, due to its cost-effectiveness, large sample size, high internal and external validity, and straightforward data analysis methods (Druckman et al., 2011). This combined approach has become a vital research tool, particularly in political science public opinion polling research (Bremer and Bürgisser, 2023; Ghassim et al., 2022) and decision-making studies in administrative management (Lee et al., 2025; Sheffer et al., 2018).

Which problems in the field of sport psychology research are appropriate for survey experiment? There are several important and unique features of survey experiment that define the applicable research areas.

The first characteristic is the large sample size, which can be as extensive as tens of thousands, an unattainable scale in a true experimental study (Sniderman, 2018). This enables the simultaneous exploration of the effects of multiple independent variables and treatment levels on the response variable. In an experimental intervention design, hundreds of treatment levels can be allocated concurrently to tens of thousands of samples. For instance, in a study on exercise adherence, numerous independent variables impact adherence, such as the time of day, economic conditions, level of education, exercise environment, skill level, and gender. Examining all these factors simultaneously helps mitigate confounding by external variables.

The second unique feature of survey experiment is that samples can be randomly selected to be broadly representative, and large random samples minimize differences between groups of participants so that even weak effects between treatments levels can be detected (Sniderman, 2018). Questionnaires are common in sport psychology research, particularly in questionnaire revisions for longitudinal follow-up studies, where framing factors, such as questionnaire wording, choice setting, and order of questions, may influence target outcomes. Unlike other independent variables that may have a significant effect on the response variable, large between-group differences can overwhelm the effect of framing. By excluding the influence of confounding variables other than the independent variable through large random samples, experimental research can more accurately observe the true direction and effect of independent variables such as the framing effect.

The third distinct feature of survey experiment is its ability to discern sequential cause-and-effect relationships through experimental design. In correlation research, many relationships are influenced by mediation through a third variable (Sniderman, 2018). Experimental research can validate genuine relationships and establish causal sequences. Within sport psychology research, distinguishing the antecedent from the consequence in mutually reinforcing phenomena poses a challenge. For instance, it is unclear whether a sports team’s group cohesion increases due to improved performance or if the team’s performance is enhanced because of increased group cohesion (Carron et al., 2002). Countless media reports have documented situations where performance and team unity have experienced simultaneous improvements or declines, resulting in a perplexing scenario. By employing a virtual case intervention, survey experimental studies enable the predetermination of cause, post-determination of effect, a reverse design, and comparison of the effects on the response variable (Sniderman, 2018).

The fourth characteristic of survey experiment pertains to the unique intervention methodology. In survey experiment research, the intervention involves presenting virtual cases to elicit judgmental responses from the participants (Sniderman, 2018). For instance, participants may be asked how they would choose to act in a hypothetical scenario. This approach allows experimental participants to refrain from directly manipulating treatments, thereby facilitating the research process and broadening the scope of the study (Druckman et al., 2011). For instance, rare events, ethically challenging experiments, and sensitive real-world scenarios can be effectively investigated using virtual cases in experimental interventions. In the realm of sport psychology, issues such as refereeing, doping, unsportsmanlike conduct, athlete aggression, fan violence, and athlete-coach relationships are well suited for survey experiment research design.

### 3.2 Design of the survey experiment

#### 3.2.1 Manipulation of variables in survey experiment

A survey experiment involves intentionally manipulating the form or layout of survey items to deduce the opinions of subjects or respondents (Mullinix et al., 2015). The term ‘experiment’ implies random assignment of subjects to control and intervention conditions, enabling a comparison of the decisions, judgements, or behaviors of subjects in both groups, thereby facilitating the discovery of cause-and-effect relationships. Based on the manipulation of variables in experimental research, three types of experiments are identified: priming experiments, factorial experiments, and list experiments (Webster and Sell, 2014).

##### 3.2.1.1 Priming design of the survey experiment

In the process of designing survey experiment, researchers have long observed that participants’ responses are influenced by the order of the choices in the questionnaire, and this order effect is a significant source of measurement error (Schiff et al., 2022). In psychological research, this order effect is referred to as the “priming effect,” where the information provided by the first option affects the respondent’s subsequent response (Ngoye et al., 2020). Analyzing subsequent responses can reveal the true opinion or attitude of the participant. One well-known priming effect is the “anchoring effect,” which is widely utilized in consumer psychology and has marked effectiveness (Furnham and Boo, 2011). Emotional priming, which has been extensively studied in psychological research, has also expanded to include negative priming effects. The methods used to induce priming effects vary and can include textual descriptions, audiovisual information, or visual stimuli, among others, particularly emotional priming, in sport psychology research (Araújo et al., 2019). For instance, when investigating the penalty bias of football referees, priming effects can be elicited by exposing referees to a website of a favorite team before asking them to determine the card to be awarded for a foul committed by a player on that team. Initiation survey experiment can illuminate participants’ inherent judgement and decision-making biases (Raab et al., 2019).

##### 3.2.1.2 Factorial design of the survey experiment

The paramount feature of survey experiment is its ability to conduct large sample random sampling at a low cost, enabling the simultaneous exploration of the effects of multiple correlated independent variables on the dependent variable (Auspurg and Hinz, 2014). In traditional experimental designs, supporting a sufficient sample size becomes challenging, and managing various levels of factors can result in intricate interpretations, potentially undermining the validity of the statistical conclusions. Conversely, a factorial design of the survey experiment can synthesize diverse levels of independent variables, embedding their interaction within different virtual cases for comprehensive depiction. Moreover, factorial design is valuable for addressing rare events or ethical violations that are impractical to replicate or implement (Treischl and Wolbring, 2022). For instance, in situations such as fan mass violence where direct on-site experimentation is unfeasible and ethical constraints prohibit realistic simulations, virtual cases can be employed to examine the concurrent impact of multiple factors on fan mass violence.

##### 3.2.1.3 List design of the survey experiment

Measuring sensitive questions poses a challenge in empirical scientific research, as respondents may opt to either withhold answers or provide untruthful responses based on subjective inclinations (Lei and Lu, 2017). In both survey research and experimental research, social expectation behaviors come into play. In laboratory experiments, this is known as the main test effect or experimental expectation effect, while in survey research, it is referred to as socially desirable bias, making it notably challenging to elicit genuine participant responses on matters pertaining to privacy or socially sensitive issues (Ried et al., 2022). Within survey experiment, list survey experiment are more straightforward and inconspicuous than randomized response designs, allowing participants to weigh various supporting factors in a virtual case without being influenced by individual question effects (Glynn, 2013). For instance, when investigating the attitudes of coaches or athletes towards doping or examining referee fairness in sport psychology, where obtaining genuine participant responses is notoriously challenging, the implementation of list design in survey experiment can help mitigate the influence of privacy or social pressure (Braga et al., 2017).

#### 3.2.2 Randomized sample allocation

According to experimental research, participants or subjects can be randomly assigned to treatment levels in three ways: between-subjects, within-subjects, and mixed designs (Keren and Lewis, 1993). In a between-subjects design, each subject is assigned only one treatment of the independent variable, also known as an independent group design. As each subject received only one treatment, there was no possibility that one treatment influenced or contaminated the other. One distinct feature of the between-subjects design is that each subject can have only one score. An advantage of the between-subjects design is that the scores are independent. Because each subject participates in only one measurement, the researcher has confidence in the purity of the results, which are uncontaminated by other treatment factors; however, a large sample size is required for a between-subjects design. The within-subjects experimental design involves comparing two or more treatment conditions (or treatments and controls) by observing and measuring the same group of subjects receiving all the treatment conditions. An essential feature of within-subjects experimental design is that it uses only one subject group, exposing them to all treatments and observing and recording their psychology and behavior across the various treatment conditions. In statistical analyses, within-subjects designs are often referred to as repeated measures designs. However, the main advantage of the within-subjects design is that it fundamentally eliminates problems arising from individual differences, which is a drawback of the between-subjects design. A mixed design combines the advantages of both between-subjects and within-subjects designs by arranging some independent variables in a between-subjects design and others in a within-subjects design. In survey experiment, the between-subjects design is more widely used due to the unique advantage of large-sample random sampling, especially in simple randomized design for list survey experiment, randomized design for factorial survey experiment, and block group randomized design and stratified randomized design for routine and initiation survey experiment in numerous cases (Bansak et al., 2021).

#### 3.2.3 Random sample selection

Random sample selection ensures a high degree of equivalence in various observable and unobservable characteristics among respondent groups and eliminates omitted variables and sample selection bias, thereby establishing a counterfactual comparison for causal inference. This comparison demonstrates that the intervention (independent variable), by stimulating and transmitting information, has a genuine causal impact on the outcome variable (dependent variable), as opposed to purely quantitative correlation. Inferential statistics derive their value and meaning from the presence of the sample-overall relationship. The representativeness of the drawn sample is a critical measure of external validity. The representativeness of the sample is determined by two factors: whether the sample is randomly selected and the size of the sample space, i.e., the sample size (Gobo, 2004). Experimental research must meet these two conditions and can use experimental design to manipulate variable treatments, thereby addressing both internal and external validity. Common probability samples used in experimental research include simple random sampling, equidistant sampling, stratified sampling, cluster sampling, systematic sampling, and others (Acharya et al., 2013). Determining the sample size primarily depends on the number of target variables in the study to calculate the minimum sample size. If the target variable is discrete, the proportional method is chosen; if it is continuous, the average method is used to calculate the estimated sample size. The specific sampling method and the determination of the sample size are also based on the random allocation method. For a between-subjects design, a slightly larger and more costly sample size is needed.

### 3.3 The application of survey experiment in sports psychology

#### 3.3.1 Design of the survey experiment: a study of sport judgement and decision-making

In Round 16 of the Champions League on the early morning of 12 March 2015, Paris Saint-Germain drew 2–2 with Chelsea in extra time, resulting in a total score of 3–3 over the two rounds. They advanced to quarter-finals based on the away goals rule. In this match, Paris Saint-Germain’s key player Ibrahimović was sent off early. At the 31st minute, Ibrahimović kicked Oscar squarely in the face. Despite Ibrahimović having retracted his feet by that point, the referee, influenced by Oscar’s exaggerated reaction, issued a red card to Ibrahimović. According to Ibrahimović, “When I saw the red card, I thought, ‘Does not this guy know what he’s doing? Maybe he saw something completely different.’ However, that was not the worst of it. The worst thing was that when I received the card, all the Chelsea players surrounded me (except for the keeper), and I felt like I had 11 babies around me.

Without pressure from the Chelsea team, the referee may not have issued a red card to Ibrahimović. Regarding whether Oscar was feigning, Ibrahimović stated, “When I saw him approaching, I had already retracted my foot. I am unsure if he was feigning afterwards or if he was genuinely kicked by me.” Following the match, Paris Saint-Germain manager Franco Branco expressed readiness to appeal Ibrahimović’s red card, whereas Chelsea manager Jose Mourinho pleaded for leniency, hoping for Ibrahimović’s continued participation in the Champions League. Moreover, the UEFA was in the process of formulating new rules to reduce the influence of player pressure and interference on referees. This incident raises questions about the impact of collective player pressure on referees’ penalty decisions in sports. The concentrated pressure from the Chelsea players, excluding the goalkeeper and the grounded Oscar, alongside the nine players surrounding the referee simultaneously, may have significantly influenced the referee’s decision to issue a red card. Humans often make impulsive decisions when influenced by their surroundings, cues, and emotions.

The priming design of survey experiment presents significant opportunities in the realm of sports judgement and decision-making (Williams and Jackson, 2019). In the penalty decision-making process within sports competitions, the referee’s rulings might elicit violent reactions from athletes. Traditional research theories propose that teams that frequently pressure the referee may gain an advantage in penalty decision-making, while conflicting studies suggest the opposite effect (Dawson and Dobson, 2010; Lane et al., 2006). It has also been reported that consistent pressure on the referee could lead to resentment and unfavorable game outcomes (Jones et al., 2002). This situates the phenomenon within the dichotomy of “the crying child will be fed” or “the crying child will be beaten.” What are the perspectives of the general public regarding this phenomenon? Furthermore, how much pressure is sufficient to prompt favorable treatment rather than adverse consequences? If such a study were conducted in a controlled environment, the limited sample size and high costs would constrain the generalizability of the findings. Additionally, the small sample size would hinder the differentiation of subtle differences between treatments when manipulating the independent variable.

*In this priming survey experiment design, we explore the impact of group pressure on penalty decision-making behavior. The experiment involved six levels of group pressure, ranging from 0 to 5. Initially, a slow-motion video of Ibrahimović’s foul on Oscar will be presented with only two people featured—the offender and the fouled—representing level 0. Subsequently, the footage will be shown sequentially with 1, 3, 5, 7, and 9 players from Chelsea’s team surrounding the referee, exerting pressure. Participants will then be asked to assess the severity of Ibrahimović’s foul in each of the six scenarios, categorizing the severity of the penalty into 10 levels and providing scores for each level.*

*In the priming survey experiment, the 6 levels of group pressure were manipulated using a between-subject experimental design, with approximately 200 subjects assigned to each treatment level. It is evident that while laboratory experiments may be prone to failure in studying initiation effects, randomized large-sample survey experiment are more appropriate for this type of research.*

In survey experiment, priming effects are classified as assimilation priming or contrast priming. The boundary between these two types of priming in the context of group sentencing decision-making influences the bias in referee sentencing decisions. An important consideration for the experimental design of priming investigations is the need for verification of the manipulation in virtual case intervention designs to ensure the desired priming effect. However, verifying the effect of emotional priming on implicit memory requires a sophisticated experimental design (Molden, 2014).

#### 3.3.2 Factorial design of the survey experiment: exercise adherence research

The issue of exercise adherence holds enduring significance in the field of sport psychology (Buckworth and Dishman, 2007). Research on exercise adherence predominantly focuses on cross-sectional surveys, while laboratory experiments are uncommon. This rarity is primarily due to the intricate nature of operationalizing target variables for exercise adherence and the multifaceted influence of individuals, environments, and tasks on exercise adherence. Additionally, the interplay among variables complicates the control of confounding effects and the isolation of causal relationships (Rhodes et al., 1999).

Both laboratory and field behavioral experiments encounter challenges in controlling for confounding effects and isolating one-to-one causal relationships. Survey research often involves descriptive studies of variable correlations, hindering the exploration of causal relationships. To address these limitations, experimental studies using a factorial design for survey experiment can incorporate multiple target variables in virtual case intervention conditions. This involves randomly assigning respondents to different versions of the questionnaire and implementing diverse intervention scenarios. By integrating the virtual case and employing a mixed experimental design, these studies can explore the causal relationships and interaction effects between the independent and dependent variables. Participants are randomly selected to explore the effects of the independent variable and the dependent variable simultaneously.

In the context of exercise adherence, the initial consideration in a factorial design of the survey experiment involved determining the independent variable. The independent variables influencing exercise adherence, based on the existing research literature and empirical theories, include free time, economic conditions, skill level, social support, exercise environment, education level, body condition, and gender (Buckworth and Dishman, 2007). Subsequently, the levels at which these variables should be measured must be determined, with an emphasis on achieving maximal conciseness while retaining more degrees of freedom. For example, time was categorized into two levels (holiday, non-holiday), economic conditions into two levels (white-collar car, blue-collar bike), skill level into two levels (teaching others to swim, self-teaching to swim), social support into two levels (accompanied, unaccompanied), exercise environment into two levels (neighborhood swimming pool, distant swimming pool), education into two levels (college, non-college), body condition into two levels (last month’s swimsuit, unfit swimsuit), and gender into two levels (male, female). Accordingly, a total of 256 dummy cases can be constructed depending on the variable type. After eliminating unreasonable combinations, such as driving a car or riding a bicycle to a nearby swimming pool, 128 valid virtual cases remained.

*Ms. Li is an employee in a white-collar company. Tomorrow, Saturday, is a holiday. She was thinking about what to do the evening of the holiday when her university girlfriend called to ask her to go to a swimming pool ten kilometers away the next night to teach her to swim. She took the swimsuit that she bought last month out the boot of the car. The swimsuit did not fit well; Ms. Li looked at herself in the mirror, in deep thought about the next night’s swimming activities.*

*If you were Ms. Li, would you go swimming? (There are 11 levels of scoring from not going swimming to going to swimming, with 5 points for going swimming, −5 points for not going swimming, and 0 points for not going swimming).*

After defining virtual cases in the factorial design of survey experiment, other crucial considerations include determining the number of response cases and respondents for each virtual case. This entails deciding the quantity of treatments and observations for each treatment. Ideally, more observations per treatment yield better results but must be balanced against participants’ cognitive load and potential confusion. It is advisable to limit respondent cases to avoid cognitive impairment, which typically do not exceed 10 cases per respondent. For instance, with 8 copies for each virtual case and 128 virtual cases, 16 case samples are generated. If each case sample requires 150 observations, the participant sample size needed is 2,500 respondents. Additionally, random selection of the 16 patients and the 2,500 respondents from the population should be considered, followed by random assignment of the respondents to the treatment groups. Analyzing the impact of virtual case level and the respondent level in experimental research with multiple independent variables and mixed designs is best suited for multilevel modelling.

#### 3.3.3 List of survey experiment: attitudes towards doping research

The issue of doping in sports endures, despite international sports organizations achieving consensus on its prohibition. Doping remains prohibited in actual competitions (Mazzeo et al., 2018). The perceptions and attitudes of coaches and athletes towards doping are critical for combating doping. However, in many research studies, coaches and athletes have not provided real answers regarding their attitudes towards doping, resulting in few truthful responses (Morente-Sánchez and Zabala, 2013). Therefore, a list survey experiment can mitigate the impact of strong social desirability effects, preventing respondents from providing untruthful answers to safeguard their personal privacy in research on this sensitive issue.

In the experimental design of listing surveys, it should be noted that the items should be negatively correlated with each other to avoid ceiling effects or floor effects in respondents’ answers. The experiment comprised four groups, including a control group, where participants were directly questioned about their attitudes towards doping and whether they exhibited socially desirable behaviors. Experimental Group 1 involved events unrelated to doping, while Experimental Group 2 included scenarios in which the athletes secretly doped themselves. In Experimental Group 3, the scenarios focused on coaches doping their athletes, with all other options described similarly to those in Experiment 1. The four treatments were as follows:

*Control Group: In the course of sports career competitions, athletes or coaches often dope to win championships, gold medals or improved results. If you were an athlete, would you secretly dope? (Yes, No, Do not know) If you were a coach, would you let athletes dope? (Yes, No, Do not know).*

*Experimental Group 1: In a sports career competition, athletes or coaches often encounter the following situations to win championships, gold medals or achievements. If you are an athlete or coach, how many of the following things might you do? Indicate how many of them you would do, without answering the specific options.*

1. *Disagree with the referee’s decision and put pressure on the referee.*
2. *Provoke an opponent verbally with the aim of having the opponent ejected from the match.*
3. *Commit tactical fouls to prevent the opponent from scoring.*
4. *Bribe the referee to sway the outcome of the game.*

*Experimental Group 2: In the course of their sports career, athletes or coaches often encounter the following situations to win championships, gold medals, or achievements. If you were an athlete or coach, how many of the following things would you probably do? You only need to indicate how many of these things you would do, not answer the specific options.*

1. *Disagree with the referee’s decision and put pressure on the referee.*
2. *Provoke the opponent verbally with the aim of having the opponent ejected from the match.*
3. *Commit tactical fouls to prevent the opponent from scoring.*
4. *Secretly dope yourself for the sake of gold medals, honors or results.*
5. *Bribe the referee to influence the result of the match.*

*Experimental Group 3: In the course of their sports career, athletes or coaches often encounter the following situations to win championships, gold medals, or achievements. If you were an athlete or coach, how many of the following things would you probably do? You only need to indicate how many of these things you would do, not answer the specific options.*

1. *Disagree with the referee’s decision and put pressure on the referee.*
2. *Provoke the opponent verbally with the aim of having the opponent ejected from the match.*
3. *Commit tactical fouls to prevent the opponent from scoring.*
4. *As a coach, make athletes take performance-enhancing drugs for the sake of gold medals, honors or results.*
5. *Bribe the referee to influence the results of the match.*

The four treatments were implemented in a between-subjects design, with 800 subjects randomly selected and divided into four groups, each consisting of 200 subjects. The participants included various populations, including athletes, coaches, and laypeople. The dependent variable involves the difference derived from subtracting the mean value of response entries, calculated separately for the control and experimental groups. Compared with direct questioning, enumeration experiments have a substantial impact on model estimation, yielding more reliable and credible analytical results (Chang and Krosnick, 2010). These survey experiments typically utilize a completely randomized design, leading to the statistical treatment of data using factorial analysis.

### 3.4 The advantages of the internet and big data in fueling survey experiment

The impact of the internet and big data on the research paradigm in sport psychology is reflected in four aspects. Leveraging the internet and big data expands the sample space indefinitely, while research costs can be more effectively managed, particularly for survey experiment, as the sample closely represents the entire population. Moreover, the experimental context aligns closely with real-life scenarios, and the data closely reflect behavioral patterns, with individual characteristics closely representative of overarching patterns.

With the advent of the internet and big data, the scope of survey experiment will expand, significantly enhancing the study’s external validity and facilitating the exploration of interaction effects between multiple variables, particularly in analyzing the causal factors in survey experiment. Furthermore, big data analysis can help mitigate complexities resulting from an excessive number of factors, leading to more conclusive research outcomes. The internet and big data greatly facilitate the accessibility and convenience of survey experiment and have been increasingly utilized in various fields, such as psychology, economics, management, political science, sociology, and particularly in public administration, where survey experiment is prevalent (Walker et al., 2017; Walker et al., 2019). The line between laboratory experiments and internet-based surveys is blurred due to the incorporation of multimedia elements, such as sound, video, photographs, and detailed graphics. It is anticipated that, in the near future, the rapid proliferation of big data and the internet will confer increasing benefits to survey experiment in the field of sport psychology.

In scientific research, internal and external validity are crucial in determining the quality of a study. Experimental studies generally offer high internal validity but suffer from lower external validity. In contrast, questionnaire surveys tend to have greater external validity while exhibiting lower internal validity. Survey experiment, by incorporating large samples and experimental controls, are able to more effectively balance both internal and external validity. Survey experiment, by utilizing large samples and experimental controls, effectively balance internal and external validity and are now widely applied in fields such as political science and social sciences. However, survey experiment has seen limited application in the area of sports psychology. Therefore, this review emphasizes the essence and features of survey experiment, its research design, and practical examples of its application in sports psychology. It is hoped that this review will increase awareness and encourage the use of survey experiment among scholars in sports psychology.

## 4 Discussions

It is challenging for commonly used experimental research and survey studies to balance internal and external validity. The literature review revealed that survey experiment integrates the advantages of survey research, including large random samples for external validity, with the benefits of experimental research, such as control and manipulation for internal validity (Martini and Olmastroni, 2021). However, there is currently no research that provides a comprehensive summary of the application of survey experiment in sports psychology. Therefore, this review aimed to address this gap by systematically reviewing the literature on the use of survey experiment in the field of sports psychology. Based on a thorough literature review, the current review addressed the following aspects: the connotations and characteristics of survey experiment; the design of survey experiment; the application of survey experiment in sports psychology; and the advantages of the Internet and big data in advancing survey experiment. We first explored the connotations and characteristics of survey experiment, and identified that the primary characteristics of survey experiment include a large sample size; the ability to randomly select samples that are broadly representative; and the capacity of large random samples to minimize differences between participant groups, thereby enabling the detection of even weak effects between treatment levels. Furthermore, the survey experiment has the ability to discern sequential causal relationships through experimental design, along with a unique intervention methodology. Subsequently, we focused on the design of survey experiment, which consists of three components: (1) Manipulation of variables in survey experiment. In this section, we elaborate on the commonly employed research designs for variable manipulation in survey experiment; (2) Randomized sample allocation; (3) Random sample selection. Then, we presented three typical cases of survey experiment applied in the field of sports psychology. Through these cases, we illustrated the application of survey experiment in the field of sports psychology. Finally, the impact of the internet and big data on the research paradigm in sports psychology is manifested in four aspects: (1) Expanding the sample space; (2) Aligning the experimental context closely with real-life scenarios; (3) Enhancing the study’s external validity and facilitating the exploration of interaction effects; (4) Helping to mitigate complexities arising from an excessive number of factors.

Since its emergence, the survey experiment research has been widely used due to its high research validity, low cost, and straightforward data processing methods, particularly in the social sciences such as political science, sociology, and economics. However, this type of research has not been performed in the field of sport psychology. Scholars in the field of exercise psychology have opted for large-sample cross-sectional research utilizing multivariate statistics and complex statistical models to increase the overall validity of the research. Internal validity is the only way to accurately reflect the causal relationships between variables; no matter how intricate the statistical models are, the causal relationships obtained without manipulating the target variables will not be reliable. This has left scholars in the field of sport psychology who prefer the natural empirical research paradigm increasingly perplexed by the ecological validity of laboratory research, particularly in the field of cognitive decision-making in sports, where the lack of connection between laboratory experiments and real-life scenarios is highly questionable. Despite the development of field experiments on sports behavioral decision-making based on the experimental research paradigm of behavioral economics, they are limited by their sample sizes and inability to randomly sample. Survey researchers are hindered by a statistical curse, while laboratory research is restricted by the manipulation of variables. Longitudinal development can only be increasingly extreme in one aspect; however, no matter how advanced the methodology is, it can never avoid the inherent issues and drawbacks of both.

The use of survey experiment research in political science, the social sciences, and other fields can be a successful approach, although it is important to be mindful of the limitations of inferring causation. Sport psychologists should assess the durability of survey experiment and, as a general rule, carry out longitudinal studies of multiple relationships. Randomized survey sequences can be used to collect most of the data and investigate potential causal links. However, contamination between experiments is unavoidable if multiple experiments are conducted. Researchers can use simple calculations to determine whether other factors influence the estimated intervention effect (Gaines et al., 2007).

Caution should be taken when making causal inferences through vignettes, and it is essential to distinguish between errors from situational experiments and those from the real world. In competitive sport psychology, game scenarios should be studied prospectively rather than retrospectively. The experimental approach is considered the ‘gold standard’ for causal inference in empirical research and has been instrumental in addressing the long-standing ‘fundamental problem’ of causal inference in sport psychology (Jackson and Cox, 2013). Despite its limitations, the survey experiment has a broad range of potential applications and is of great value in the field of sport psychology and kinesiology. Advances in computer technology have enabled the use of virtual case interventions in various forms, such as video, pictures, and sound, while the internet has increased the reach of participants and lowered the cost of surveys. Additionally, big data have made it easier to collect large sample sizes, and technological advances have been instrumental in promoting and popularizing survey experiment paradigms. Therefore, sport psychology research, whether focused on the humanities and social sciences or natural empirical research, should take advantage of the changing research methodology and lead the development of sport psychology research methodology.

Although this exploratory review summarizes the characteristics, research designs, and potential applications of survey experiment in sport psychology, certain limitations persist. First, relatively few of the included studies involve survey experiment in the field of sport psychology, which is largely due to the limited application of survey experiment in this area to date. Therefore, we suggest that future research should adopt survey experiment in sport psychology. Second, given that this review employs a narrative approach, the future studies may consider conducting meta-analyses when the application of survey experiment becomes increasingly prevalent in the field. This approach can more intuitively highlight the advantages of survey experiment over traditional experimental research and questionnaires in terms of internal and external validity.
